# Identification and genetic characterization of equine infectious anemia virus in Western Balkans

**DOI:** 10.1186/s12917-021-02849-2

**Published:** 2021-04-15

**Authors:** Diana Lupulovic, Sara Savić, Delphine Gaudaire, Nicolas Berthet, Živoslav Grgić, Kazimir Matović, Alexandre Deshiere, Aymeric Hans

**Affiliations:** 1 Scientific Veterinary Institute “Novi Sad”, Rumenacki put 20, Novi Sad, 21000 Serbia; 2 ANSES- Laboratory for Animal Health in Normandy, Physiopathology and Epidemiology of Equine Diseases Unit, Goustranville, France; 3 The Center for Microbes, Development and Health, CAS Key Laboratory of Molecular Virology and Immunology, Institut Pasteur of Shanghai – Chinese Academy of Sciences, Discovery and Molecular Characterization of Pathogens, Shanghai, China; 4 Institut Pasteur, Unité Environnement et Risque Infectieux, Cellule d’Intervention Biologique d’Urgence, Paris, France; 5 Chinese Academy of Sciences, Shanghai, 200031 China; 6 Specialist veterinary institute Kraljevo, Zicka 34, Kraljevo, Serbia

**Keywords:** Equine infectious anemia, Horses, NGS, AGID test

## Abstract

**Background:**

Equine infectious anemia (EIA) is a viral disease, caused by the Equine Infectious Anemia virus (EIAV) belonging to the *Retroviridae* family, genus *Lentivirus*. Horses (or equids) infected with EIAV are lifelong carriers and they remain contagious for other horses even in the absence of clinical signs. So far, EIAV infection has been reported among horses in North and South America, France, Germany, Italy, Hungary and Romania, with no publication regarding the presence of EIAV in horses in Serbia. To determine the circulation of EIAV among, approximately, the 5000 horses of the Vojvodina region, northern part of Serbia, 316 serum undergone serological testing for EIA. Then, identification and full genome sequencing using next generation sequencing was performed from one EIA positive horse.

**Results:**

the 316 sera were tested with 3 different commercial agar gel immunodiffusion (AGID) tests and two different commercial enzyme-linked immunosorbent assay (ELISA). With the three AGID kits, 311 (98.4%) among the 316 tested sera were negative and only five (1.6%) sera were positive for EIA. Some discrepancies were seen for the two ELISA kits tested since one exhibited the same results as AGID test and the second gave 295 sera with negative results, five with a positive result and 16 with doubtful outcome. Phylogenetic analysis performed using the full genome sequence showed that EIAV characterized from a horse in Serbia is different from those identify so fare around the world and form a distinct and separate group together with another EIAV strain.

**Conclusions:**

This study demonstrate for the first time that EIAV is circulating at a low level in the horse population from the Northern part of Serbia. Interestingly, phylogenetic data indicates that this EIAV from the western Balkan region of Europe belongs to a new cluster.

## Background

Equine infectious anemia (EIA) is a persistent viral infection of equidaes. The causative agent, EIA virus (EIAV) belongs to the genus *Lentivirus* from the *Retroviridae* family, subfamily *Orthoretrovirinae*. Genus *Lentivirus* also includes Human immunodeficiency virus (HIV), Bovine and Feline immunodeficiency viruses (BIV and FIV) as well as the Visna-maedi virus. Infected horses are lifelong carrier of the virus and remain contagious for other horses even without overt clinical signs. EIAV, a blood borne virus, is transmitted from one animal to another usually by hematophagous insects or iatrogenically through contaminated needles or dentistry equipment. Bloodsucking insects – primarily horse flies and stable flies – are mechanical vectors. Although the virus does not replicate within the insect, the virus remains infectious in its mouthparts for several hours after a bite. Equine infectious anemia has been reported in the USA, Canada, Latin America, Europe and Asia and affects horses, ponies, mules, and donkeys [1–7].

Pathogenesis of the disease is highly variable, reflecting a wide range of clinical forms of the disease – from unapparent infection to death. Once the horse is infected, several clinical forms are possible. Acute form of the disease is often associated with the primary infection and clinical symptoms include pyrexia, anorexia, depression, and petechial bleeding of the mucosa. Anemia is not typical as acute infection, except in very severe cases, in which epistaxis and ventral oedema may occur. Death of the animal can occur within 4 weeks after the primary infection. If the animal survives the acute phase, the frequency and the severity of the clinical episodes progressively drops, until the animal becomes an unapparent reservoir of the virus. Nevertheless, in the fields, some horses serologically positive to EIAV, have never manifested any clinical symptoms, or they were in such a mild form to remain unnoticed by owners. Diagnostics of EIA relies on the detection of antibodies against viral components and antibodies to EIAV can be detected using enzyme-linked immunosorbent assay (ELISA) and agar gel immunodiffusion (AGID) test. In Serbia, there is an annual monitoring system of the disease, carried out by the *Ministry of Agriculture*, *Forestry and Water Management*, *Veterinary Directorate*. According to this program, each horse, registered in Serbia, has to be tested for EIA once a year. However, horses used for the production of biological materials, such as hyperimmune sera or vaccines, have to be tested twice a year. Moreover, when the owner is selling or on any other way alienating the animal, they have to present a certificate, not older than 30 days, stating that the horse is negative for EIA. The main serological diagnostic assay used in Serbia for EIAV testing is AGID test and, in parallel, ELISA test may be used but should be confirmed by an AGID test in case of positive finding. Equine infectious anemia is a notifiable disease in Serbia and all EIAV positive horses are reported to Veterinary Directorate and OIE. EIA positive horses are euthanized according to the Serbian legislation. The aim of this study is to report, for the first time, the seroprevalence of EIAV in Serbian horses and its molecular characterization using next generation sequencing of EIAV of horses from Vojvodina region (Northern part of Serbia).

## Results

### Serological results

**Fig. 1:**
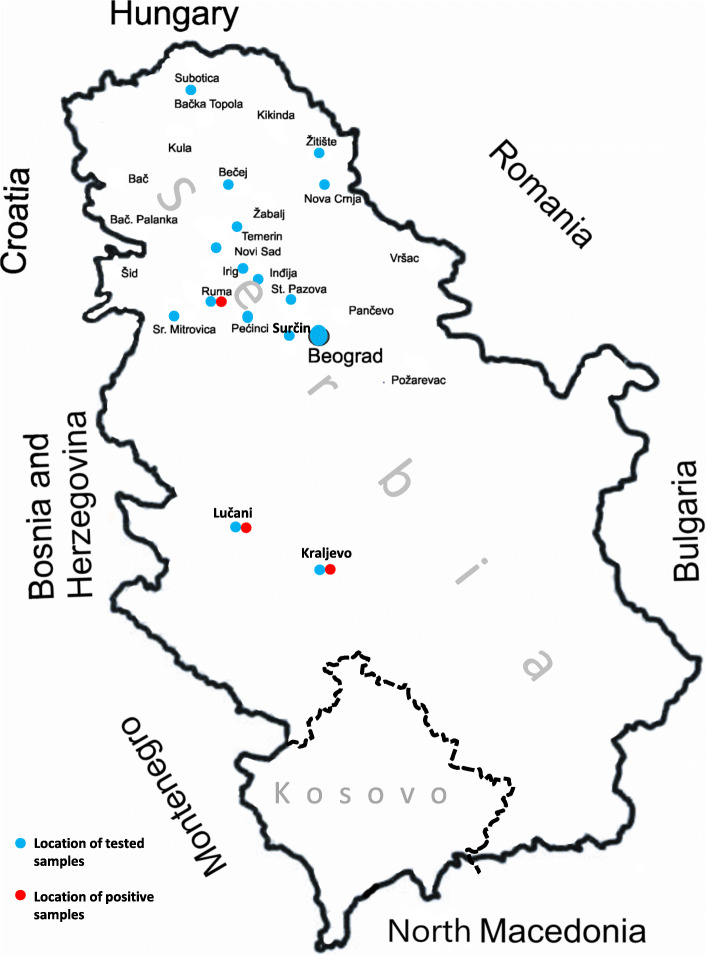
Geographic distribution of collected samples between 2013 and 2014 that have been tested for equine infectious anemia infection in the Northern part of Serbia. The map is an own creation

Between 1994 and 2013, a total of 11,972 horses serum samples were analysed using AGID tests. The obtained results show that 21 horses were found positive for EIA in the region of southern Backa and Srem in Serbia. The percentage of EIA positive horses in this 20-year period represents only 0.17% of the tested horses. Another study was undertaken to validate the use of some ELISA tests, under field conditions, compared to the AGID kit used routinely in the Serbian laboratory from VMRD company. For this purpose, 316 samples collected during two-year period (2013–2014) in the northern part of Serbia (Fig. 1) were tested first with three different commercial AGID kits (Idexx, ID-Vet and VMRD). The results obtained with the three AGID kits were the same with 311 out of 316 sera negative for EIA and only 5 (1.6%) samples positives for EIA. Those results indicated that the three commercial AGID kits tested exhibit the same outcome. In a second step, the 316 serum samples were also tested with 2 commercial ELISA kits (Synbiotics and VMRD). The ELISA kit from Synbiotics was able to find the same results as the AGID kits previously tested. However, the VMRD ELISA kit gave 295 sera with negative results, 5 with a positive results and 16 with doubtful outcome. Those results indicate that the two ELISA kits tested in this study could be used in diagnostic laboratories given that all positive or doubtful samples in ELISA should be retested and confirmed in AGID test as previously described [8].

### Virus genotyping

**Fig. 2:**
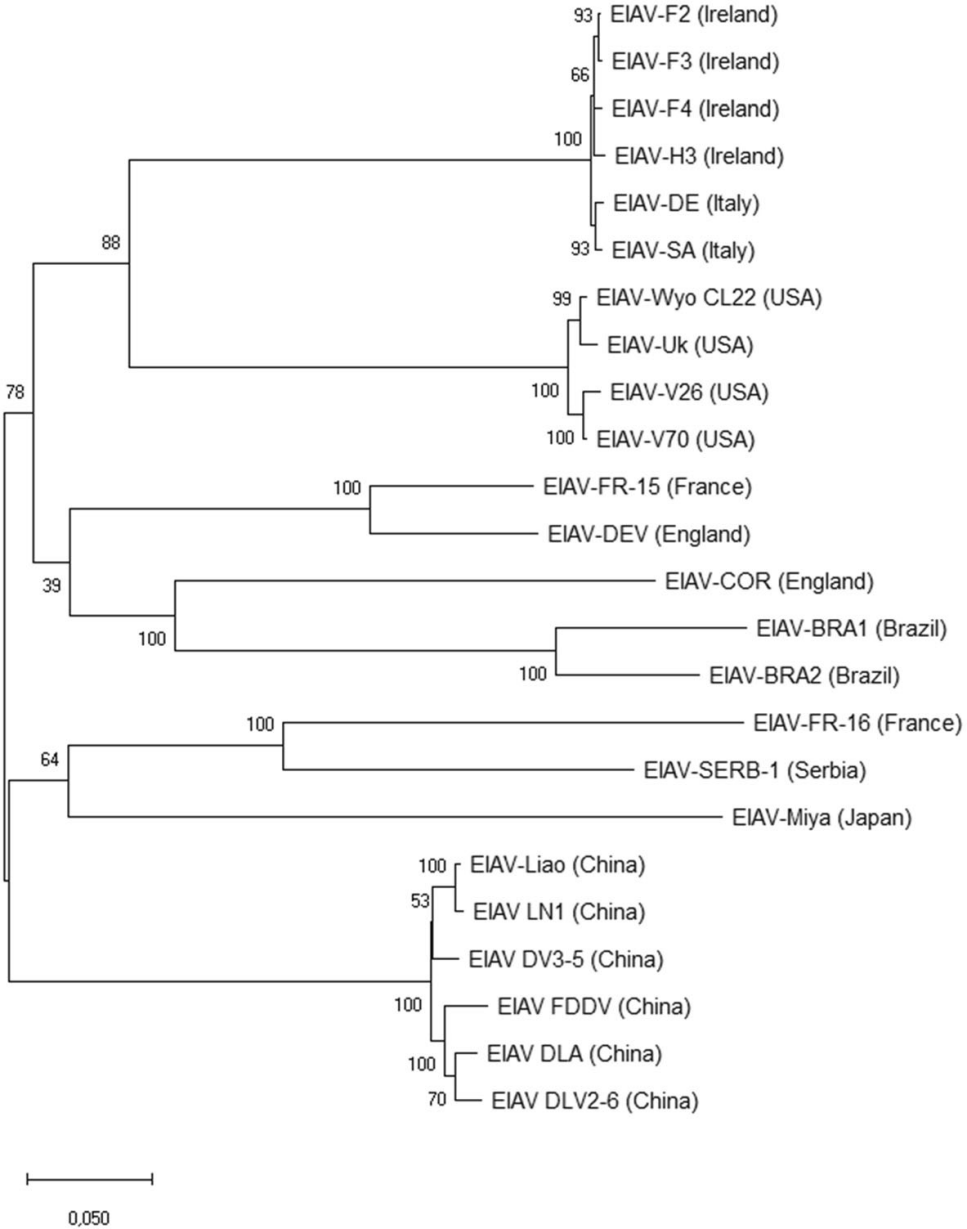
The evolutionary history was inferred by using the Maximum Likelihood method based on the Tamura-Nei model. The percentage of trees in which the associated taxa clustered together is shown next to the branches. The analysis involved 24 EIAV full genome sequences. All positions containing gaps and missing data were eliminated. There were a total of 7269 positions in the final dataset. Evolutionary analyses were conducted in MEGA7

Furthermore, the spleen of one horse out of 5 found positive between 2013 and 2014 was able to be collected after euthanasia. The full genome sequence of the EIA virus (EIAV-SERB-1) from this horse was performed using the targeted sequence enrichment and next generation sequencing as described previously [9]. This sequence was compared to the 23 full genome sequences of EIAV published so far in the literature from England (EIAV-COV and EIAV-DEV), USA (Wyo, Uk, V26 and V70), Japan (EIAV-Miya), Ireland (F2, F3, F4 and H3), Italy (SA and DE), France (EIAV-FR-15 and FR-16), China (Liao, LN1, DV3–5, FDDV, DLA and DLV2–6) and Brazil [9–13]. Interestingly, the phylogenetic tree (Fig. 2) revealed that EIAV-SERB-1 form a new branch with the strain EIAV-FR-16 isolated in 2014 in France from an asymptomatic Friesian stallion. The nucleotides comparison study indicates that this two EIA isolates, EIAV-SERB-1 and EIAV-FR-16, share 78.7% of identity. However, epidemiological investigation did not established any relationship between those two cases.

## Discussion

The outbreaks statements in the north part of Serbia over the last 20 years (1993–2013) were rather sporadic, without cases declared during some years, and with only few EIA positive horses diagnosed each time. Those results seem to indicate that EIA does not widespread in the Vojvodina region of Serbia which has border with Romania, which is known as endemic country for EIAV.

Moreover, we observed that the 24 EIAV isolates fully sequenced and described so far in the scientific literature can be divided into 6 different clades or groups. One branch represents EIAV characterized from outbreaks declared in Ireland and Italy in 2006, a second branch is composed of viruses coming from the USA which include the Wyoming reference strain. Two more branches of the phylogenetic tree represent viruses isolated from horses in China and in Japan. The two last branches are composed from EIA viruses recently characterized in France, England and Serbia. Those results indicate that EIAV isolates can be divided, at least, in 6 distinct clades, based on the full EIAV genome sequences, which do not represent the geographical origin of the infected horses since 3 of them are composed of viral isolates retrieved from horses held in Europe.

## Conclusions

In conclusion, the data presented in this study indicate that EIA is present in the horse population of Serbia at a low level, certainly thanks to the surveillance program implemented by Serbian authority since 1981 [14]. This low EIA occurrence allows the use of ELISA test to facilitate the screening test for the annual EIA surveillance program in Serbia given that all positive sample with ELISA should be confirmed with AGID test. Moreover, our findings reinforce the large diversity of EIAV isolates characterized around the world during the last decade and it is the first study to demonstrate the presence of EIAV in Serbia associated to the full genome sequence characterization using NGS.

## Methods

### Blood samples

A total number of 11,972 horse sera samples, collected from 1994 to 2013, were analyzed for EIA using agar gel immunodiffusion test (AGID) as described by the World Organization of Animal Health (OIE) chapter 2.5.6. The samples of horses analysed were from two different regions of Serbia southern Backa and Srem. Sera samples from 316 horses, held in the Province of Vojvodina in Serbia, were collected between 2013 and 2014.

### Serological testing with AGID and ELISA kits

Sera have been tested with 3 different commercial AGID test (Idexx, ID-Vet and VMRD) following manufacturer’s instructions in two different laboratories (ANSES-Laboratory for animal health which is the European Reference laboratory for equine diseases other that African sickness and Scientific Veterinary Institute “Novi Sad”, Department for serology, immunology and biochemistry) and two different commercial ELISA tests (Synbiotics and VMRD) as recommended by manufacturer.

### Spleen sample, viral genome sequencing and phylogenetic analysis

Horses were subject to euthanasia as enforce by the Serbian legislation as follow, an intravenous injection of the anaesthetic Xilazin 2% was performed. The usual dose of Xilazin for anaesthesia is 1,1 mg/kg, so for a horse that weight 500 kg half a dose would be 12-15 ml of Xilazin 2% just for sedation. The horse became very quiet in several minutes. Then a mix of euthanizing substances (T61) was injected intravenously to the EIA infected horses. For a horse of 500 kg it should be a dose of 50 ml. After 20–30 s, the horse just kneeled down and then slowly lied to the side. There was no excitation, no nervous reactions. Once the death is confirmed by the official veterinarian, spleen from one EIA positive horse was collected and kept at − 80°c until use. Genomic DNA from collected spleen was extracted as previously described [4]. DNA libraries were prepared using the SureSelect XT Library preparation kit for Illumina Multiplexed sequencing Kit (Agilent Technologies, Santa Clara, CA, USA) as described previously [9] and subjected to multiplex deep sequencing on the MiSeq platform using the MiSeq Reagent Kit V2 (Illumina). Fastq format files from each viral genome were trimmed and paired reads were merged using the Geneious Prime sequencing data analysis software (Biomatters LTD, Auckland, New Zealand) [15]. Contigs were obtained by alignment to the reference genome EIAV F2 (GenBank accession number: JX480631.1). Phylogenetic reconstruction was carried out using the MEGA software version 7 [16]. Nucleotide and amino acid identities were calculated using MegAlign from DNA star software.

## Data Availability

The datasets used and/or analysed during the current study are available from the corresponding author on reasonable request. Sequence from this study has been deposited in NCBI Genbank under the following accession number MT 338937.

## Abbreviations

AGID: Agar Gel ImmunoDiffusion
BIV: Bovine Immunodeficiency Virus
EIA: Equine Infectious Anemia
EIAV: Equine Infectious Anemia Virus
ELISA: Enzyme-linked ImmunoSorbent Assay
FIV: Feline Immunodeficiency Virus
HIV: Human Immunodeficiency Virus
OIE: World organization for animal health
USA: United States of America
